# Surplus Photosynthetic Antennae Complexes Underlie Diagnostics of Iron Limitation in a Cyanobacterium

**DOI:** 10.1371/journal.pone.0018753

**Published:** 2011-04-20

**Authors:** Paul S. Schrader, Allen J. Milligan, Michael J. Behrenfeld

**Affiliations:** Department of Botany and Plant Pathology, Oregon State University, Corvallis, Oregon, United States of America; University of Florida, United States of America

## Abstract

Chlorophyll fluorescence from phytoplankton provides a tool to assess iron limitation in the oceans, but the physiological mechanism underlying the fluorescence response is not understood. We examined fluorescence properties of the model cyanobacterium *Synechocystis* PCC6803 and a Δ*isiA* knock-out mutant of the same species grown under three culture conditions which simulate nutrient conditions found in the open ocean: (1) nitrate and iron replete, (2) limiting-iron and high-nitrate, representative of natural high-nitrate, low-chlorophyll regions, and (3) iron and nitrogen co-limiting. We show that low variable fluorescence, a key diagnostic of iron limitation, results from synthesis of antennae complexes far in excess of what can be accommodated by the iron-restricted pool of photosynthetic reaction centers. Under iron and nitrogen co-limiting conditions, there are no excess antennae complexes and variable fluorescence is high. These results help to explain the well-established fluorescence characteristics of phytoplankton in high-nutrient, low-chlorophyll ocean regions, while also accounting for the lack of these properties in low-iron, low-nitrogen regions. Importantly, our results complete the link between unique molecular consequences of iron stress in phytoplankton and global detection of iron stress in natural populations from space.

## Introduction

Proliferation of oxygenic photosynthesis approximately 2.3 billion years ago dramatically reduced iron solubility in the surface ocean and created a ‘physiological crisis’ for phytoplankton that continues today [1]–[5]. Photosynthesis is an iron-demanding process, with a single copy of the linear electron transport chain requiring ∼24 atoms of iron [6]. Accordingly, phytoplankton exhibit a variety of plastic and constitutive changes in their photosynthetic membranes in response to low iron levels [7]–[10]. These responses create signals that allow detection of iron stress in the field and, by far, the most commonly exploited signal is changes in chlorophyll fluorescence properties.

The fraction of absorbed light lost as fluorescence increases under low iron conditions, which is indicative of decreased efficiency in energy transfer for photosynthesis. This response is so universal and readily observed that it has served as the defining physiological diagnostic of iron stress in the field [11]–[16]. Yet despite its great utility, molecular mechanisms underlying this fluorescence response are not well understood, in part due to a mismatch between typical laboratory iron-starvation conditions and the low-iron, steady-state growth conditions prevalent in the open ocean. To address this issue, we conducted experiments with the model cyanobacterium, *Synechocystis* sp PCC 6803, under three steady-state nutrient regimes: (1) nitrate and iron replete, (2) limiting-iron and high-nitrate, representative of natural high-nitrate, low-chlorophyll (HNLC) regions, and (3) iron and nitrogen co-limiting, simulating conditions found in regions of the central Pacific gyres [15], [17]. With this system, we successfully reproduce fluorescence properties observed in the field under low iron conditions, link these properties to previously resolved macromolecular structures induced under iron stress, and identify significant physiological interactions between micronutrients and macronutrients that modify the fluorescence response.

*Synechocystis* is a common cyanobacterium in fresh water systems that has a sequenced genome and, due to the availability of genetically modified strains, has become a model organism for photophysiological research. Iron-stress has been thoroughly studied in *Synechocystis*, with a dominant response being the degradation of normal peripheral light harvesting antennae (phycobilisomes) and replacement with new light harvesting supercomplexes around PSI that are composed of multiple monomers of the chlorophyll-a binding protein encoded by *isiA* [18]–[20]. While a comprehensive review of its cellular function is beyond the scope of this study, IsiA is reported in the literature to increase the absorption cross section of PSI and to provide protection from high light, oxidative stress, salt stress, and heat stress [6], [21]–[32].

Appearance of IsiA supercomplexes provides a sensitive physiological signal of iron stress in *Synechocystis*, while availability of a Δ*isiA* knock-out mutant [33] allows clear distinction of fluorescence changes associated with peripheral antennae. Thus, despite being a freshwater species, *Synechocystis* proffers significant advantages for resolving fundamental mechanisms of iron-stress physiology. To evaluate the broader relevance of our findings, we compare our laboratory results to fluorescence properties of natural iron-stressed phytoplankton populations in the Pacific Ocean.

## Results and Discussion

**Figure 1:**
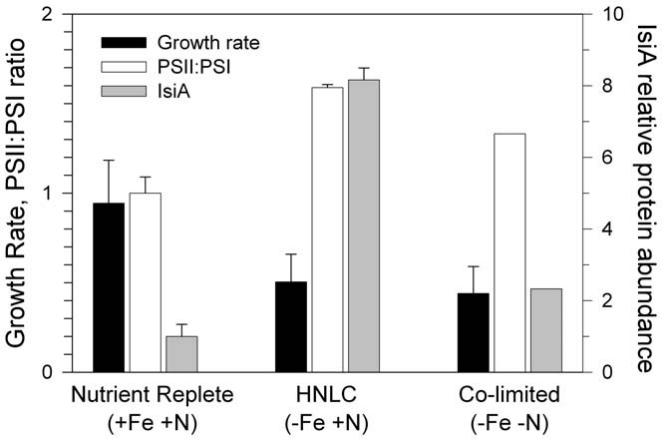
Comparison of growth rates and protein abundance. Response of wild type *Synechocystis* sp. PCC 6803 growth rate (d^−1^), PSII∶PSI ratio (normalized to nutrient replete conditions) and IsiA abundance (normalized to background density value) for the three steady-state nutrient conditions (labelled at bottom). Error bars are standard deviations of duplicate cultures.

In all our *Synechocystis* experiments, cultures were maintained at steady state growth for a minimum of 7 generations prior to harvesting. The steady-state specific growth rate of nutrient-replete *Synechocystis* was 0.9 d^−1^, with no detectable expression of IsiA protein (Fig. 1). Our iron-limiting and nitrate-replete treatment reduced specific growth rates to 0.5 d^−1^, consistent with previous laboratory and field-based iron-limited growth rates [34] (Fig. 1). In this simulation of natural HNLC conditions, iron limitation caused a 60% increase in the PSII∶PSI ratio and an 8-fold increase in IsiA protein abundance (Fig. 1), consistent with previous iron-starvation results [19], [20], [35], [36].

In a similar manner as the HNLC condition, iron and nitrate co-limitation caused a 33% increase in PSII∶PSI and enhanced levels of IsiA, relative to the nutrient replete condition. These responses are clear indications of iron stress (Fig. 1). However, IsiA was 4-fold lower in the co-limiting treatment than in the HNLC simulation, demonstrating a strong interaction between iron- and nitrate in expression of this pigment-protein (see Figure S1 for raw Western Blot data). Furthermore, the nearly equivalent growth rate for these two treatments suggests that IsiA abundance is in excess of growth requirements when external nitrate is plentiful (i.e., HNLC conditions). Electron microscopy shows that these excess IsiA supercomplexes lack PSI reaction centers [21], [37].

The existence of antennae complexes in excess of reaction center requirements is anticipated to have significant consequences on fluorescence properties. To test this prediction, we used a Fast Repetition Rate fluorometer (FRRf) [38] to routinely monitor initial (F_o_) to maximal (F_m_) fluorescence induction curves. Traditionally, fluorescence measured at ambient temperature is assumed to originate predominantly from chlorophyll associated with PSII, while the contributions from PSI are assumed negligible due to the high efficiency of PSI energy transfer. Accordingly, the value of F_o_ is viewed as an index of total fluorescence emission when all primary electron acceptors of PSII (Q_A_) are functionally ‘open’ (i.e., oxidized) and available for photochemistry, while the enhanced value of F_m_ is a measure of fluorescence yield when all Q_A_ are reduced and functionally ‘closed’. The normalized difference between F_m_ and F_o_ [i.e., F_v_/F_m_ = (F_m_−F_o_)/F_m_] is a commonly used measure of the efficiency with which absorbed light energy is used for PSII photochemistry. This interpretation however, will be invalid if other chlorophyll containing proteins are significant sources of fluorescence, such as excess antennae complexes lacking PSI reaction centers. The presence of such complexes would reduce F_v_/F_m_. Importantly, one of the hallmarks of iron-limitation in the field is a depression in F_v_/F_m_ [11]–[15], [39].

**Figure 2:**
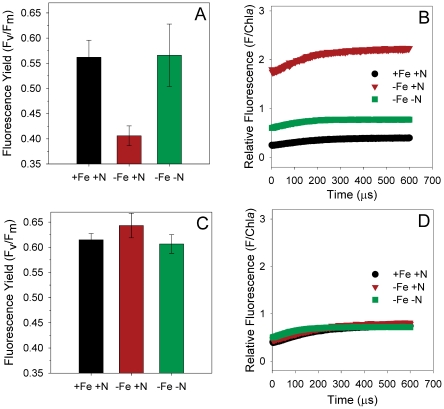
Comparison of fluorescence values. Normalized variable fluorescence yield (F_v_/F_m_) and fluorescence normalized to chlorophyll-a (ng) for the wild type *Synechocystis* sp. PCC 6803 (A and B respectively) and Δ*isiA* mutant (C and D, respectively) under each nutrient condition.

Nutrient-replete, wild-type *Synechocystis* had an F_v_/F_m_ value of 0.58±0.02 (Fig. 2A). HNLC conditions caused a decrease in F_v_/F_m_ to only 0.40±0.01 that resulted from a pronounced increase in overall fluorescence yield (Fig. 2A,B). This observation is again consistent with elevated nitrate leading to over-expression of IsiA complexes with high fluorescence emission. In contrast, co-limited *Synechocystis* exhibited fluorescence and F_v_/F_m_ values equivalent to nutrient-replete conditions (Fig. 2A,B), indicating efficient energy transfer between IsiA and PSI (i.e., no excess antennae complexes). Furthermore, we find that all three nutrient regimes have equivalent fluorescence properties in the Δ*isiA* mutant (Fig. 2C,D). These results clearly demonstrate the central role of over-expressed peripheral IsiA antennae complexes on the enhanced fluorescence emission and depressed F_v_/F_m_ in wild-type *Synechocystis* under HNLC conditions.

**Figure 3:**
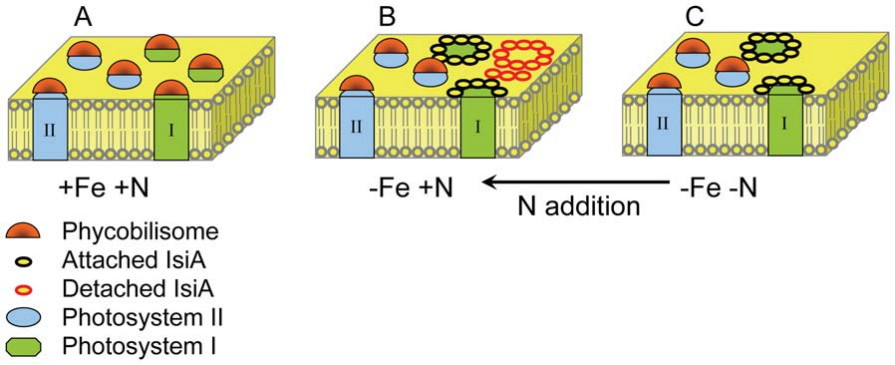
Alterations in photosynthetic architecture of thylakoid membranes driven by nutrient availability. A) Under nutrient replete conditions, variable fluorescence is high and both PSII and PSI have phycobilin-based light harvesting complexes (phycobilisomes). B) Under iron limitation and plentiful nitrogen, PSI declines, phycobilisomes serve only PSII, and IsiA light harvesting complexes are expressed in excess of PSI requirements (shown as empty rings). High fluorescence yields from these empty complexes drive variable fluorescence down. C) Under iron limitation and low nitrogen, IsiA is expressed but not in excess of PSI. These complexes have efficient energy transfer and variable fluorescence is similar to nutrient replete conditions. Both in the laboratory and field, addition of nitrogen to co-limited cells drives the system to over-express antennae (indicated by arrow).

Results from our experiments help resolve an outstanding enigma regarding iron stress. Earlier measurements of energy transfer from IsiA supercomplexes to PSI indicate very high efficiencies and minimal fluorescence emission [36]. In contrast, iron-limited phytoplankton are typically associated with high total fluorescence emission and low light utilization efficiency [14], [39]–[41]. By simulating both HNLC and co-limiting conditions, we find that a single mechanism can account for these apparently incompatible earlier results. Specifically, peripheral antennae complexes (in this case IsiA supercomplexes) are produced at levels closely matching reaction center abundance when iron is limiting and nitrate is low. In this case, energy transfer efficiency to reaction centers is high and fluorescence properties are indistinguishable from nutrient replete conditions (Fig. 3). In contrast, iron stress in the presence of elevated nitrate interferes with the balanced production of antennae and reaction centers, giving rise to two distinct populations of complexes: (1) antennae associated with reaction centers and having high energy transfer efficiencies and (2) antennae energetically decoupled from reaction centers and having high fluorescence emission (Fig. 3). Recently, electrophoretic mobility shift assays have shown that iron and nitrogen regulons are present upstream of *isiA* [42], providing support for an interactive iron-nitrogen regulatory system and a starting point for resolving details of this interaction.

**Figure 4:**
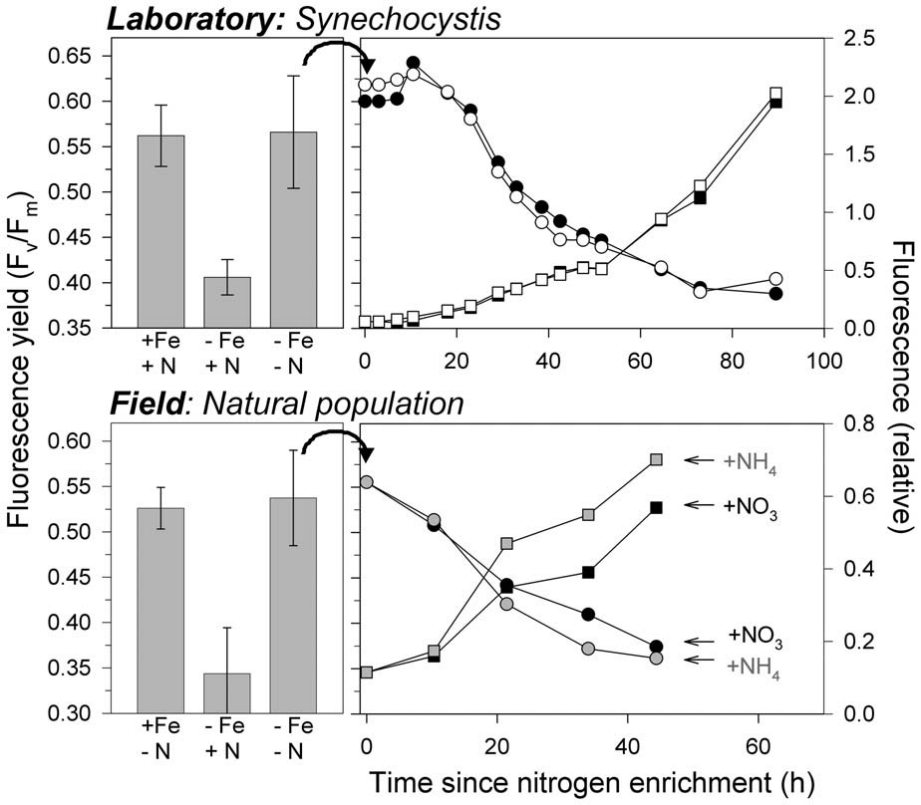
Comparison of laboratory experiments on wild type *Synechocystis* (top) with analogous experiments using mixed populations in the field (bottom). Left panels show F_v_/F_m_ for three nutrient conditions: (left) replete-iron, (center) iron-limiting (HNLC), and (right) nitrogen and iron co-limiting. Right panels show time courses of change in F_v_/F_m_ (circles, left axis) and initial fluorescence, F_o_, (squares, right axis) following nitrogen addition to co-limited populations (equivalent to converting a co-limiting environment into HNLC conditions). For *Synechocystis* (top right), open and closed symbols show results for replicate experiments. For field experiments (bottom right), black symbols show results for nitrate addition and grey symbols show results for ammonium addition. This experiment was conducted on September 8, 2002 at 7°N, 146°W and is representative of observed responses for all 6 enrichment experiments in co-limiting waters (see Methods).

In addition to assessing steady-state physiological characteristics under the three nutrient conditions, we also evaluated the temporal response of fluorescence properties to nitrogen enrichment in co-limited *Synechocystis* (Fig. 4). Transition from the co-limited state (high F_v_/F_m_, modest IsiA abundance, high PSII∶PSI) to the HNLC state (low F_v_/F_m_, greatly increased IsiA abundance, high PSII∶PSI) was complete within approximately 80 h (Fig. 4, top right panel). Nitrate addition resulted in a roughly exponential rise in fluorescence and decrease in F_v_/F_m_. These responses to nitrate addition in *Synechocystis* cells expressing iron-stress-induced IsiA and elevated PSII∶PSI verify that co-limitation was successfully achieved during our study, and they also enable a more in-depth comparison with experimental data from the open ocean.

To extend our findings to natural environments, we compare fluorescence properties and nutrient addition responses of *Synechocystis* to those observed in the field. Earlier, we conducted an extensive, 10-year survey of fluorescence properties in surface phytoplankton populations across the equatorial Pacific HNLC region and north Pacific gyre [15]. This work identified three physiological regimes of nutrient status: limiting nitrogen and sufficient iron, limiting iron and excess nitrogen (HNLC), and co-limiting iron and nitrogen (equivalent to the three conditions used in the current study). As with *Synechocystis*, only the low-iron, high-nitrogen regime in the Pacific exhibited suppressed F_v_/F_m_ (Fig. 4, bottom left panel). Moreover, addition of nitrogen had the same effect in co-limited natural populations as in *Synechocystis*. Specifically, a boost in either nitrate or ammonium increased cellular fluorescence and decreases F_v_/F_m_ (Fig. 4, bottom right panel). Recognizing that *isiA*-containing cyanobacteria comprise, at best, only a fraction of the natural population biomass, this consistency in fluorescence behaviour may suggest that over-expression of light-harvesting antennae under HNLC conditions is a more general physiological phenomenon.

A variety of responses to iron stress appear to be highly conserved across algal taxa, including an overall down-regulation of photosynthetic electron transport components, a stoichiometric shift in reaction centers toward higher PSII∶PSI ratios, and replacement of cytochrome c*_553_* and ferredoxin with plastocyanin and flavodoxin, respectively [7]–[10]. Whether over-expression of antennae under HNLC conditions is another shared response remains to be tested. Nevertheless, modifications in antennae pigment-binding proteins under iron stress appear common, whether involving normal- or special iron-induced structures [11]–[15], [39]. For example, a homolog of *isiA* occurs in the cultured marine cyanobacterium, *Synechococcus* sp. CC 9605, and additional homologus genes are found in the Atlantic, Pacific, and Indian Oceans [43] (Figure S2 and Figure S3). Prochlorophytes, such as the marine phytoplankton *Prochlorococcus*, do not have a unique iron-stress antennae protein, but their normal protein (PcbB) closely resembles IsiA and is dramatically upregulated in some species during iron stress to form enlarged ring structures around PSI akin to the IsiA supercomplex [44]. In eukaryotic alga, an iron-stress induced pigment-protein (Tidi) has been identified in the halophilic organism *Dunaliella salina* and performs a similar role as IsiA [45]. An analogous chlorophyll-binding protein upregulated under iron limitation has also been suggested in marine diatoms [46].

Diagnostic fluorescence properties have been employed on ocean basin scales to map distributions of iron limited phytoplankton [15], but without complete understanding of the underlying physiology. Here, a model phytoplankton species, an ideal mutant, and realistic nutrient environments are used to elucidate a key mechanism accounting for the differential impacts of iron stress in high- and low- nitrate waters. Our findings, however, raise a question regarding the potential physiological or evolutionary value of overexpressed pigment-protein complexes under HNLC conditions. Without function, it would seem that such excess should be selected against. One possible explanation relates to the nature of iron availability in the upper ocean. Aeolian dust deposition is an important, but episodic source of new iron. Accordingly, excess pigment-protein complexes could serve as a rapid response system to iron pulses, allowing stored pigment to be quickly assimilated into functional reaction center antennae. Laboratory iron enrichment experiments showing increases in reaction center absorption cross sections in the presence of pigment synthesis inhibitors support this concept of mobilizing chlorophyll reserves of the excess pigment-protein complexes [47].

Results of our study may also shed light on an intriguing new finding regarding satellite measurements of surface chlorophyll fluorescence. Analysis of these data reveals a global correspondence between elevated phytoplankton fluorescence yields and anticipated regions of iron stress [16]. During the current study, we detected a pool of over-expressed pigment-protein complexes with fluorescence yields that were roughly an order of magnitude greater than those of functional pigment-protein complexes (see File S1). Such decoupled complexes could, in part, provide a mechanistic explanation for the enhanced fluorescence yields observed from space in low-iron regions.

The existence of over-expressed chlorophyll-containing complexes under HNLC conditions has an additional broad-scale ramification: not all chlorophyll is photosynthetically active. Satellite-derived chlorophyll data are regularly used to calculate global ocean primary production, with generally no distinction between active and inactive chlorophyll. However, Behrenfeld et al [15] proposed that a pool of dissociated chlorophyll exists under HNLC conditions that must be accounted for in primary production calculations. They used field measurements of variable fluorescence to estimate this pool as representing up to half of the total chlorophyll stock. Our culture studies support this initially crude assessment. We again found that roughly half of the total chlorophyll under HNLC conditions existed in uncoupled IsiA complexes. This finding further supports the differential treatment of HNLC waters in global assessments of ocean primary production.

## Materials and Methods

### Ethics Statement

There are no relevant permits or permissions required for international waters where field measurements were taken.

### Laboratory Experiments

#### Strain and Growth Conditions

*Synechocystis* sp. PCC 6803 wild type and Δ*isiA* knockout mutant strain cells were grown at 30°C in YBG-11 [48] medium bubbled with filter sterilized compressed air, under constant illumination at 50 µmole photons m^−2^ s^−1^ in 1 L water-jacketed glass chambers. Cultures were first acclimated and then grown under steady-state conditions for at least 7 generations. Cell enumeration was performed using a Coulter particle counter (Model 3, Beckman). Chlorophyll-*a* was determined on cells filtered onto GF/F (Whatman) glass fiber filters, extracted with 100% methanol for 2 hours with periodic shaking, and absorbance measured spectrophotometrically [49].

Culture medium was prepared using strict trace-metal clean techniques under class 100 flowhood conditions. The concentration of available iron was controlled both by initial iron concentrations and chelator concentration (EDTA). The equilibrium concentration of metal species were calculated using Visual MINTEQ ver. 2.6 [50]. For iron replete medium, 6 µM Fe and 16 µM EDTA were added to give a free ferric ion concentration of 2.40×10^−10^ M. For iron-limiting medium, 18 nM Fe and 28.4 µM EDTA were added to give an unchelated iron concentration, Fe′, of 8.07×10^−14^ M. In iron-limiting medium, steady state growth rates were 0.5 d^−1^. For both high and low iron cultures, biomass was kept at a constant level of approximately 3×10^6^ cells mL^−1^ by diluting cultures with fresh medium (17.5 mM nitrate) supplied from a peristaltic pump. To obtain iron-nitrogen co-limited cultures, fresh medium with 175 µM nitrate was added to the culture using a peristaltic pump according to the equation
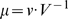
(1)where μ is the intrinsic rate of increase in biomass (d^−1^), *v* is the added volume of fresh medium per day (ml) and *V* is the volume of the culture (ml). Co-limitation was achieved by using the same iron concentrations from the iron-limiting medium (18 nM) and a series of experiments in chemostats with progressively lower nitrate concentrations (1.75 mM–175 µM until a steady-state growth rate similar to that from iron-limitation alone was achieved and maximum variable fluorescence returned to levels observed in iron-replete conditions. Co-limitation was verified for iron by expression of IsiA and elevated PSII∶PSI and for nitrogen by observing fluorescence responses to nitrate addition (see main text).

#### Protein Collection, SDS-PAGE and Western Blot Analysis

Thylakoid membrane proteins were collected as previously described [51]. A cocktail of protease inhibitors (Roche Applied Science) was added to the extracted proteins that were subsequently stored in liquid nitrogen. Protein was quantified spectrophotometrically using a BCA kit (Pierce) and chlorophyll concentration was analyzed as above. Protein (5 µg per lane) was loaded on 4-15% precast linear gradient polyacrylamide gels (Bio-Rad) run in standard Tris/Glycine/SDS buffer [52]. The separated proteins were transferred to a nitrocellulose membrane probed by antibody – IsiA, PSII (CP43) or PSI-A (all from Agrisera) and detected by chemiluminescence (Pierce – Super Signal West Pico). Samples from each replicate experimental treatment were prepared at the same time and run on identical gels under identical conditions for Western Blot analysis with the various antibodies listed above.

#### Fast Repetition Rate fluorometer (FRRf) Measurements

Measurements of F_o_, F_m_ and F_v_/F_m_ were performed using a custom benchtop Fast Repetition Rate fluorometer. Samples were dark adapted for three minutes and exposed to brief saturating light flashes (0.7 µs flash, 1.5 µs darkness for 600 cycles) to measure the fluorescence from fully oxidized (F_o_) to fully reduced (F_m_) PSII reaction centers. To ensure that PSII reaction centers were fully reduced, 10 µM DCMU [3-(3,4-dichlorophenyl)-1,1-dimethylurea], an inhibitor of electron transfer from Q_A_ to Q_B_, was added to determine F_m_ [53].

### Field Studies

Field measurements were conducted between 1994 and 2006 in the equatorial and subtropical north Pacific ocean. During this period, 25 nutrient enrichment experiments were conducted, along with continuous measurements of surface phytoplankton fluorescence properties across >58,000 km of ship transect lines. A complete description of results from these studies is provided in Behrenfeld et al. 2006. In situ nutrient conditions for the 25 enrichment experiments were determined from measured nutrient concentrations, dawn values of F_v_/F_m_, and the extent of nocturnal F_v_/F_m_ decrease [15]. Fifteen of the 25 enrichment experiments were conducted in high-nutrient, low-chlorophyll (HNLC) waters. Six experiments were conducted in Fe and N co-limiting waters. Four experiments were conducted in iron-replete waters. For each experiment, initial fluorescence properties were compared with continuous underway data to ensure consistency. F_v_/F_m_ values shown in the bottom left panel of Figure 4 in the manuscript are mean values (plus standard deviations) for all experiments conducted in the three nutrient regimes encountered during the field study.

#### Field Nutrient Enrichment Experiment Methods

Uncontaminated seawater was pumped onboard ship through a Teflon-lined polyethylene tube (lowered to ∼10 m at each station) using an air-powered polypropylene body, Teflon diaphragm deck pump, directly into a HEPA filtered laminar flow bench. All surfaces contacting seawater were rigorously cleaned using trace-metal grade 1% HCl, followed by rinsing with deionized water (Milli-Q) and with ∼60 L of seawater before sampling began. Unfiltered trace-metal clean seawater was dispensed into 10 L acid-washed carboys and either unaltered (i.e., control) or nutrient enriched. For the nitrogen enriched treatments, carboys were innoculated with either 5 µM NO_3_ or 5 µM NH_4_. Samples were then incubated at ambient surface temperature and under non-photoinhibitory light levels [15]. Subsamples were collected for the subsequent 48 hours and immediately analyzed with the FRRf, with triplicate measurements made for each treatment. Incubation carboys were maintained in the light or dark (depending on time of day) prior to sample collection for FRRf analysis to avoid any short-term changes in photosynthetic parameters. At the end of each incubation experiment, 500 ml samples were collected from each treatment carboy and analyzed for chlorophyll concentration. Chlorophyll samples were gently filtered through Whatman GF/F filters, which were then placed in glass scintillation vials with 10 ml of 90% acetone and stored in a freezer for 24 to 36 h. Chlorophyll concentration, was determined from the acetone extracts using a calibrated Turner Designs fluorometer (Model 10AU). Observed responses to nutrient enrichments were dramatically different *between* the three regimes (ie., +Fe/−N, −Fe/+N, and −Fe/−N), but were always consistent *within* a given regime [15]. For additional details on experimental methods or other nutrient enrichment treatments, see Behrenfeld et al 2006.

## Supporting Information

Figure S1 **Digital scan of SDS-PAGE Western Blots.** Anti IsiA-, PSII-, and PSI- antibodies were used to assess the relative protein levels in iron- and nutrient-replete cultures, iron-limited cultures and co-limited cultures.
(TIF)

Figure S2 **Phylogenetic analysis of BLAST search in GOS database.** A maximum-likelihood phylogenetic tree constructed from both reference sequences and sequences obtained by BLAST searching the GOS database with the *Synechococcus* CC9605 *isiA* gene. The tree was rooted from the CP43 ‘outgroup’ with bootstrap values displayed at the nodes in red with a cutoff value of 70. The reference sequences in the tree are coded as follows: *Prochlorococcus marinus* CP43 - CP431, *P. marinus* CP43 - CP432, *P. marinus* CP43 - Cp433, *P. hollandica* CP43 - CP434, *Thermosynechococcus elongates* CP43 - CP435, *Synechocystis* sp PCC 6803 CP43 - CP436, *Synechococcus* CP43 - CP437, *Chamydomonas reinhardtii* CP43 - CP438, *Spinacia oleracea* (Spinach) CP43 - CP439, *T. elongates* IsiA - IsiA1, *Synechocystis* sp PCC 6803 IsiA - IsiA2, *F. muscicola* IsiA - IsiA3, *Anabaena* IsiA - IsiA4, *Synechococcus* CC9605 IsiA - IsiA5, *Synechococcus* BL107 PcbD IsiA - IsiA6, *Synechococcus* 9311 PcbD IsiA - IsiA7, *Synechococcus* 9902 PcbD IsiA - IsiA8, *P. marinus* PcbB - PcbB1, *P. marinus* PcbB1 - PcbB2, *P. marinus* PcbB2 - PcbB3, *P. hollandica* PcbB - PcbB4.
(TIF)

Figure S3 **Map of IsiA in the ocean.** Locations where homologous gene sequences of the iron-stress-induced chlorophyll binding protein *isiA* (orange points) were sampled during the Global Ocean Survey (black points) conducted by the J. Craig Venter Institute. Background map shows annual 2007 chlorophyll distributions retrieved from the Sea-viewing Wide Field-of-view Sensor (*SeaWiFS*).
(TIF)

File S1 **Supporting information.**
(DOC)
