# Supplementary figures and images for: Surplus Photosynthetic Antennae Complexes Underlie Diagnostics of Iron Limitation in a Cyanobacterium
Source: PLoS One. 2011 Apr 20;6(4):e18753. doi: 10.1371/journal.pone.0018753 (PMC3080375; doi:10.1371/journal.pone.0018753)

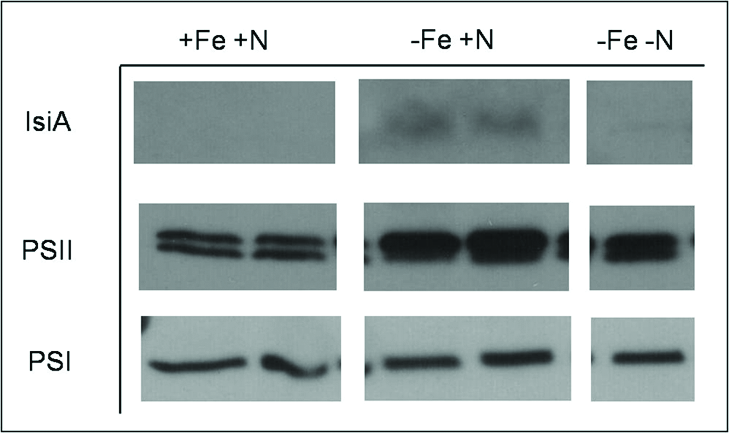

Supplement: Figure S1 — Digital scan of SDS-PAGE Western Blots. Anti IsiA-, PSII-, and PSI- antibodies were used to assess the relative protein levels in iron- and nutrient-replete cultures, iron-limited cultures and co-limited cultures. (TIF) [file pone.0018753.s001.tif]

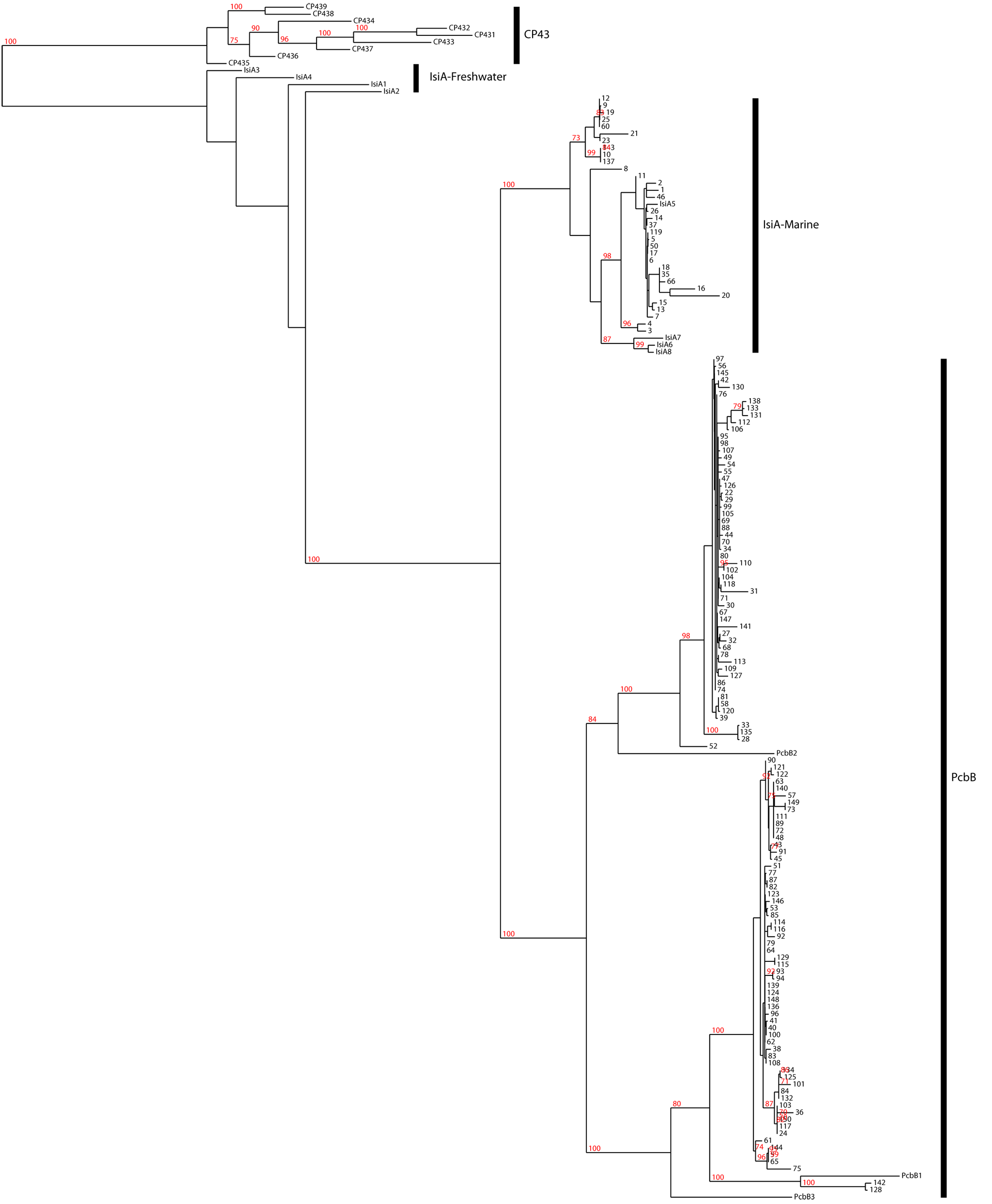

Supplement: Figure S2 — Phylogenetic analysis of BLAST search in GOS database. A maximum-likelihood phylogenetic tree constructed from both reference sequences and sequences obtained by BLAST searching the GOS database with the Synechococcus CC9605 isiA gene. The tree was rooted from the CP43 ‘outgroup’ with bootstrap values displayed at the nodes in red with a cutoff value of 70. The reference sequences in the tree are coded as follows: Prochlorococcus marinus CP43 - CP431, P. marinus CP43 - CP432, P. marinus CP43 - Cp433, P. hollandica CP43 - CP434, Thermosynechococcus elongates CP43 - CP435, Synechocystis sp PCC 6803 CP43 - CP436, Synechococcus CP43 - CP437, Chamydomonas reinhardtii CP43 - CP438, Spinacia oleracea (Spinach) CP43 - CP439, T. elongates IsiA - IsiA1, Synechocystis sp PCC 6803 IsiA - IsiA2, F. muscicola IsiA - IsiA3, Anabaena IsiA - IsiA4, Synechococcus CC9605 IsiA - IsiA5, Synechococcus BL107 PcbD IsiA - IsiA6, Synechococcus 9311 PcbD IsiA - IsiA7, Synechococcus 9902 PcbD IsiA - IsiA8, P. marinus PcbB - PcbB1, P. marinus PcbB1 - PcbB2, P. marinus PcbB2 - PcbB3, P. hollandica PcbB - PcbB4. (TIF) [file pone.0018753.s002.tif]

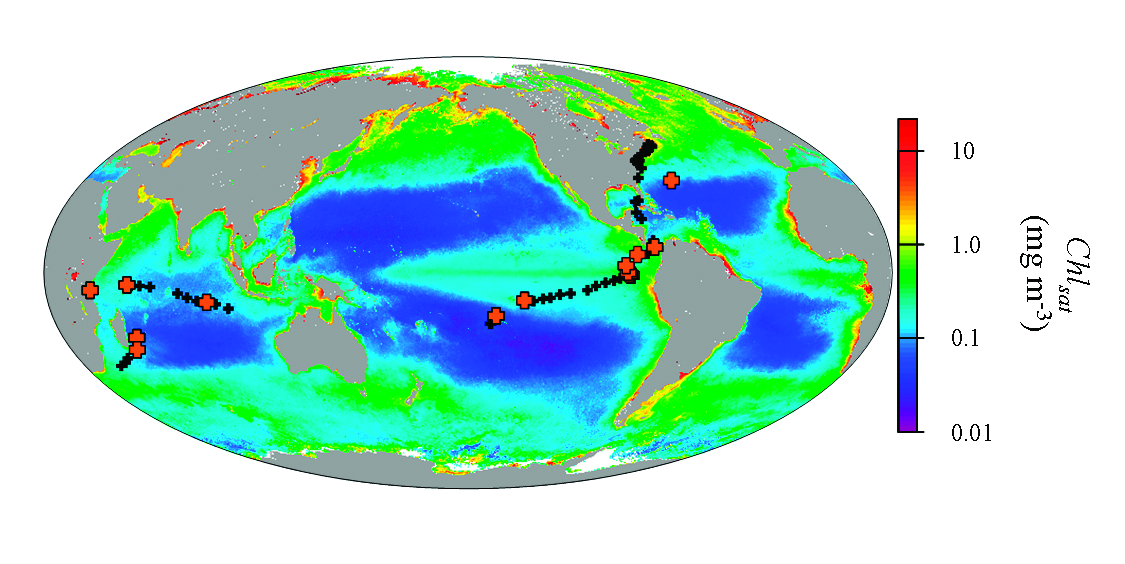

Supplement: Figure S3 — Map of IsiA in the ocean. Locations where homologous gene sequences of the iron-stress-induced chlorophyll binding protein isiA (orange points) were sampled during the Global Ocean Survey (black points) conducted by the J. Craig Venter Institute. Background map shows annual 2007 chlorophyll distributions retrieved from the Sea-viewing Wide Field-of-view Sensor (SeaWiFS). (TIF) [file pone.0018753.s003.tif]
