# Supplementary material for: Surplus Photosynthetic Antennae Complexes Underlie Diagnostics of Iron Limitation in a Cyanobacterium
Source: PLoS One. 2011 Apr 20;6(4):e18753. doi: 10.1371/journal.pone.0018753 (PMC3080375; doi:10.1371/journal.pone.0018753)
Supplement: File S1 — Supporting information. (DOC) [file pone.0018753.s004.doc]

**Supporting Information**

***BLAST and Phylogenetic Analysis***

To determine the frequency of *isiA* in field samples, we utilized the genomic database from The Community Cyberinfrastructure for Advanced Marine Microbial Ecology Research and Analysis (CAMERA) collected during the J. Craig Venter Institute’s Global Ocean Sampling (GOS) expedition [1]. We BLAST searched the metagenomic open reading frame peptide database using the IsiA sequence from *Synechococcus* sp CC9605 with a cut-off of 1E-10. The sequences were aligned using both ClustalW and T-Coffee. The alignments were refined manually based on the known sequence gap present in IsiA but absent in the closest related photosystem II protein CP43. Sequences that were too short to cover the sequence gap between CP43 and IsiA were removed. Phylogenetic trees were created using the Randomized Axelerated Maximum-Likelihood (RAxML) method on the Cyberinfrastructure for Phylogenetic Research (CIPRES) cluster at the San Diego Supercomputing Center with WAG and Gamma analysis using 1,000 replicates to give bootstrap values [2][3].

***Calculating a Relative Quantum Yield of Fluorescence for Dissociated IsiA***

Behrenfeld et al. (2009) has shown that iron-limited areas of the ocean have high fluorescence yields and suggest that this is due in part to light harvesting antennae complexes that are not functionally attached to reaction centers. Because the fluorescence detector is not calibrated in our Fast Repetition Rate fluorometer, we cannot calculate the quantum yield of fluorescence (emitted photons normalized to absorbed photons) but can calculate the increase in fluorescence from dissociated complexes relative to fluorescence from associated chlorophyll. We focus our analysis on the HNLC and dual-limited treatments. The difference in cellular Chl-*a* between these treatments is a combination of IsiA expression and growth rate. We first made the well supported assumption that Chl-*a* is a linear function of growth rate under steady state growth conditions [4, 5] and corrected the dual-limited treatment Chl-*a* values for the slight difference in specific growth rates between the HNLC and dual-limited conditions (0.50 and 0.44 respectively). This correction increases the cellular Chl-*a* and F_o_ about 15% in the dual-limited treatment. The observation that F_v_/F_m_ is the same between control and dual-limited treatments indicates that Chl-*a* in the dual-limited treatment is all associated with reaction centers. Accordingly, the chlorophyll normalized Fo for dual-limited cells is 0.29. To determine the fraction of F_o_ per cell associated with functional PSII reaction centers in the HNLC treatment, we used the following equation:

(F_decoup_ x Chl_diff_) + (F_Dual_ x Chl_Dual_) = F_HNLC_ (1)

Where:

F_decoup_ is the fraction of F_o_ per chlorophyll from decoupled PSII reaction centers

Chl_diff_ = 2.24-0.91 = 1.33 is the difference in chlorophyll (ng) per cell (HNLC – dual-limited treatments)

F_Dual_ = 0.29 is F_o_ per chlorophyll for the dual-limited treatment calculated above

Chl_Dual_ = 0.91 is chlorophyll (ng) per cell for the dual-limited treatment

F_HNLC_ = 3.86 is the total F_o_ per cell for the HNLC treatment

Solving for F_decoup_ gives a value of 2.7. The relative increase in quantum yield of fluorescence for dissociated IsiA:chlorophyll complexes is then F_decoup_ F_Dual_ gives a value of 9.5.

This is a first approximation of the relative quantum yield and does not account for the changes in PSII:PSI ratios. Here we have assumed that there are compensating changes in the absorption cross section that would mitigate the changes in reaction center stoichiometry. We have also not ruled out the possibility that some of the fluorescence could come from dissociated phycobillisome pigments. Violation of these assumptions does not change the observation that HNLC conditions produce highly fluorescent cells.

**References for Supporting Information**

1. Seshadri R, Kravitz SA, Smarr L, Gilna P, Frazier M (2007) CAMERA: A Community Resource for Metagenomics. PLoS Biol 5: e75.

2. Miller M, Holder M, Vos R, Midford P, Liebowitz T, et al. The CIPRES Portals. URL:http://www.phylo.org/sub_sections/portal: CIPRES Available at: http://www.phylo.org/sub_sections/portal.

3. Stamatakis A, Hoover P, Rougemont J (2008) A Rapid Bootstrap Algorithm for the RAxML Web-Servers. Syst. Biol. 75: 758-771.

4. Laws EA, Bannister T (1980) Nutrient-limited and light-limited growth of Thalssiosira fluviatilis in continuous culture with implications for phytoplankton growth in the ocean. Limnol. Oceanogr. 25: 457-473.

5. Halsey KH, Milligan AJ, Behrenfeld MJ (2010) Physiological optimization underlies growth rate-independent chlorophyll-specific gross and net primary production. Photosynth. Res. 103: 125-137.
